# Highly time-resolved 4D MR angiography using golden-angle radial sparse parallel (GRASP) MRI

**DOI:** 10.1038/s41598-022-18191-y

**Published:** 2022-09-05

**Authors:** Adam E. Goldman-Yassen, Eytan Raz, Maria J. Borja, Duan Chen, Anna Derman, Siddhant Dogra, Kai Tobias Block, Seena Dehkharghani

**Affiliations:** 1 Department of Radiology, Children’s Healthcare of Atlanta, Atlanta, GA USA; 2 Department of Radiology and Imaging Sciences, Emory University, Atlanta, GA USA; 3 Department of Radiology, NYU Langone Health, New York, NY USA; 4 Department of Radiology, New York–Presbyterian Hospital, New York, NY USA; 5 Department of Radiology, Maimonides Medical Center, New York, NY USA; 6 Department of Neurology, NYU Langone Health, New York, NY USA; 7 Center for Biomedical Imaging, New York University Langone Health, 660 First Ave, 2nd Floor, New York, NY 10016 USA

**Keywords:** Medical research, Magnetic resonance imaging, Neurological disorders

## Abstract

Current dynamic MRA techniques are limited by temporal resolution and signal-to-noise penalties. GRASP, a fast and flexible MRI technique combining compressed-sensing, parallel imaging, and golden-angle radial sampling, acquires volumetric data continuously and can be reconstructed post hoc for user-defined applications. We describe a custom pipeline to retrospectively reconstruct ultrahigh temporal resolution, dynamic MRA from GRASP imaging obtained in the course of routine practice. GRASP scans were reconstructed using a custom implementation of the GRASP algorithm and post-processed with MeVisLab (MeVis Medical Solutions AG, Germany). Twenty consecutive examinations were scored by three neuroradiologists for angiographic quality of specific vascular segments and imaging artifacts using a 4-point scale. Unsubtracted images, baseline-subtracted images, and a temporal gradient dataset were available in 2D and 3D reconstructions. Distinct arterial and capillary phases were identified in all reconstructions, with a median of 2 frames (IQR1-3 and 2–3, respectively). Median rating for vascular segments was 3 (excellent) in all reconstructions and for nearly all segments, with excellent intraclass correlation (range 0.91–1.00). No cases were degraded by artifacts. GRASP-MRI obtained in routine practice can be seamlessly repurposed to produce high quality 4D MRA with 1–2-s resolved isotropic cerebrovascular angiography. Further exploration into diagnostic accuracy in disease-specific applications is warranted.

## Introduction

Time-resolved magnetic resonance angiography (TR-MRA) permits non-invasive assessment of cerebral hemodynamics, including for the diagnosis and surveillance of arteriovenous shunting (e.g. from arteriovenous malformations (AVM) and dural arteriovenous fistulae (AVF)) without reliance on ionizing radiation, iodinated contrast, or cerebrovascular catheterization^1^. Existing techniques such as *Time-Resolved Imaging of Contrast KineticS* (TRICKS) and *Time-resolved angiography With Stochastic Trajectories* (TWIST) leverage data under-sampling with view sharing, parallel-imaging acceleration, and non-cartesian k-space trajectories. While beneficial for undemanding applications, such as isolating peak tissue or vascular enhancement phases, these techniques are limited to temporal resolutions of several seconds per whole-brain volume, and hence lack specificity for high-flow shunting^1–10^. Signal-to-noise penalties inherent to conventional under-sampling and acceleration strategies further impose limitations to the attainable spatial and temporal resolution of TR-MRA, which reduces its utility for characterization of small shunts. To overcome these limitations, advanced techniques have been proposed that combine 3D acceleration with a priori assumptions on the data structure (i.e., redundancy or sparsity) for achieving highly accelerated angiography, such as *Highly constrained Projection Reconstruction* (HYPR). However, HYPR requires multiple discrete and lengthy acquisitions, which increases complexity of the acquisition workflow and thus far has precluded widespread clinical adoption^11,12^. Compressed-sensing techniques are an alternative approach for increasing temporal resolution, which incorporate a priori information into the reconstruction algorithm to cope with data under-sampling. Initial applications have shown promising results^13^, particularly when combined with non-cartesian readout schemes. Clinical standard non-contrast time-of-flight (TOF) MRA is, by comparison, insensitive to flow within the venous circulation, provides no unambiguous indication of hemodynamic phases, and has been shown to be suboptimal for assessment of the angioarchitecture of cerebral AVM/AVF^12,14^.

*Golden-angle RAdial Sparse Parallel* (GRASP) is a relatively new technique for fast and flexible dynamic contrast-enhanced MRI that combines compressed sensing, parallel imaging, and golden-angle radial k-space sampling^15^. GRASP allows for continuous, rather than sequential, data acquisition and thus straightforward, user-defined post hoc reconstructions that can be optimized for application-specific use. Because radial sampling is inherently robust to motion, initial applications focused on dynamic visceral imaging to enable free-breathing examination of patients unable to suspend respiration. Further, because varying parenchymal enhancement phases could be reconstructed retrospectively from the continuously acquired data, the technique mitigates errors resulting from inaccurate timing of scans, hence simplifying clinical workflow significantly^16–20^.

While initial applications of GRASP used low temporal resolutions targeted for use in visceral imaging, we have observed that the continuous and uniform golden-angle radial sampling is particularly well-suited to capturing more rapid dynamic processes, owing to its potentially sub-second temporal resolution. Moreover, it relaxes the demand for precise synchronization of the bolus injection with sampling of the k-space center because each acquired k-space line covers the k-space center. By reconstructing the continuously acquired data with extreme under-sampling, volumes with a narrow temporal footprint can be generated for any time point within the acquisition window. Naturally, the lesser the data incorporated into the reconstruction, the larger the costs of reduced signal and the greater the potential vulnerability to artifacts. Nevertheless, we posit that the compressed-sensing reconstruction with robust *temporal total-variation* (TV) regularization and the strong sensitivity to gadolinium-induced T1 shortening are conditioned well to assessing dynamic cerebrovascular processes^15^. While past approaches have leveraged radial and other non-cartesian readouts with compressed sensing and golden-angle ordering to achieve uniform sampling, the application to large or real-world cohorts has yet to be fully realized^21–23^. The flexibility of GRASP as a technique easily implemented in routine practice, by comparison, allows for complementary TR-MRA reconstruction from data that is routinely acquired for volumetric T1-weighted structural imaging due to the high robustness to motion.

In this study we aim to demonstrate this possibility of utilizing GRASP for fully retrospective TR-MRA in a cohort of patients undergoing structural GRASP brain imaging in routine clinical practice. We hypothesize that GRASP bares untapped potential for simple and robust TR-MRA, which can be realized through a few clinically practicable post-processing steps. We present a custom reconstruction and visualization pipeline for creating multi-planar, subtracted, and time-resolved 1 mm isotropic whole brain MR angiography with 1–2 s temporal resolution.

## Methods

### Study subjects

After approval by the institutional review board (IRB) of NYU Langone Medical Center with waiver of informed consent, a retrospective query of the radiological information system was performed to identify subjects > 18 years of age with reportedly normal brain MRI obtained in routine practice with intravenous contrast. At our institution, GRASP is prescribed in standard brain MRI protocols during the first pass of gadolinium with reconstruction of 6–10 nominally time-resolved, post-contrast structural brain series, separated by ~ 20–25 s per volume depending upon the protocol. Immediately following the acquisition, raw k-space data from GRASP scans are exported to an external processing server within the hospital network, where retrospective GRASP processing can be performed using an offline reconstruction framework (Yarra Framework, https://yarra-framework.org). Parameters for the retrospective reconstruction, such as the number of radial views per volume (see below), can be selected using a graphical user interface under direction of the examiner.

All exams were obtained on clinical 3 T whole-body scanners (MAGNETOM Vida, MAGNETOM Trio, or MAGNETOM Skyra, Siemens Healthineers, Erlangen, Germany) using combined head/neck RF receive coils. A weight-based gadolinium dosing regime was employed using a high relaxivity agent (Gadobutrol, 0.1 mmol/kg) injected at 4 ml/s and followed by a normal saline flush, which corresponds to the injection rate used at our institution for routine imaging. No a priori assumptions with respect to contrast kinetics or cerebral hemodynamics, nor modifications to the injection or acquisition protocol were made in any case. GRASP data were acquired using a fat-saturated 3D gradient-echo pulse sequence with slab-selective RF excitation and radial stack-of-stars sampling of k-space according to the golden-angle scheme^24^. The contrast agent was injected 20 s after the start of the sequence. Following sequence parameters were used for the scans: TR 3.65 ms; TE 1.73 ms; flip angle 12°; pixel bandwidth 630; radial views 600; field-of-view 256 mm; base resolution 1.0 mm × 1.0 mm × 2.0 mm, reconstructed to 1.0 mm isotropic voxels; slices 170–208 per slab (interpolated); slabs 1; total acquisition time 3:20. Exact acquisition parameters, including TR and slices per slab, varied slightly due to use of different MR systems and requirement for full anatomic coverage of the brain in all patients.

After initial screening of formal radiologic interpretations to identify reportedly normal exams, the studies were reviewed by a second-year neuroradiology fellow under the supervision of a subspecialty certified neuroradiologist with > 10 years of experience in neurovascular imaging. Cases with structural abnormalities, masses, developmental anomalies, or prior injury were excluded from analysis. Additional exclusions included the absence of full GRASP data sets or missing GRASP raw k-space data, failed gadolinium injections, and examinations unrecoverably corrupted by artifacts such as from orthodontic hardware or extrinsic devices and leads. The electronic medical record was reviewed to identify any unanticipated diagnoses or abnormalities in the final clinical interpretation that might affect cerebral hemodynamics (e.g., stroke, heart failure, peripheral vascular/atherosclerotic disease, etc.).

To preliminarily assess the potential utility of GRASP MRA in the setting of intracranial pathology, subjects with intracranial AVF or AVM and undergoing routine perioperative digital subtraction angiography (DSA) contemporaneous with their MRI (within 3 months) were identified.

The study was performed in accordance with the Declaration of Helsinki.

### MRA processing

Angiographic GRASP images were reconstructed retrospectively on an external processing server using a C/C++ implementation of the GRASP algorithm. Reconstructions used 5 radial views per frame, producing an effective frame rate in the range of 1.58–2.07 s per whole 3D brain volume, depending on the acquisition parameters TR and number of slices. Using an Ubuntu Linux server with two Intel Xeon E5-2698 processors, a total of 32 cores, and 256 GB memory, the average reconstruction time per case was 3 h. Subtractions and further post-processing steps of the reconstructed images were performed using a custom pipeline implemented in a publicly available DICOM processing toolbox (MeVisLab, MeVis Medical Solutions AG, Bremen, Germany, https://www.mevislab.de) running on a separate workstation. Briefly, GRASP images were converted to the compact MeVisLab format to facilitate file handling and post-processing and were made available for review as contiguous, multi-planar reformatted (MPR) dynamic 2D slices and 3D rendered maximum intensity projections (MIP) with and without baseline subtraction in all cases. Baseline frames were selected by the user, typically between the 16th–18th temporal frame to ensure a clean steady state of the signal. In order to better isolate the parenchymal blood pool in the capillary phase and to recapitulate catheter-angiographic tissue hemodynamic signatures, dynamic images were also separately processed in MeVisLab by calculating the gradient of the subtraction images with respect to the time dimension and displaying the magnitude value of the gradient, which accentuates temporal changes in the subtraction images.

### Image review and rating

**Figure 1:**
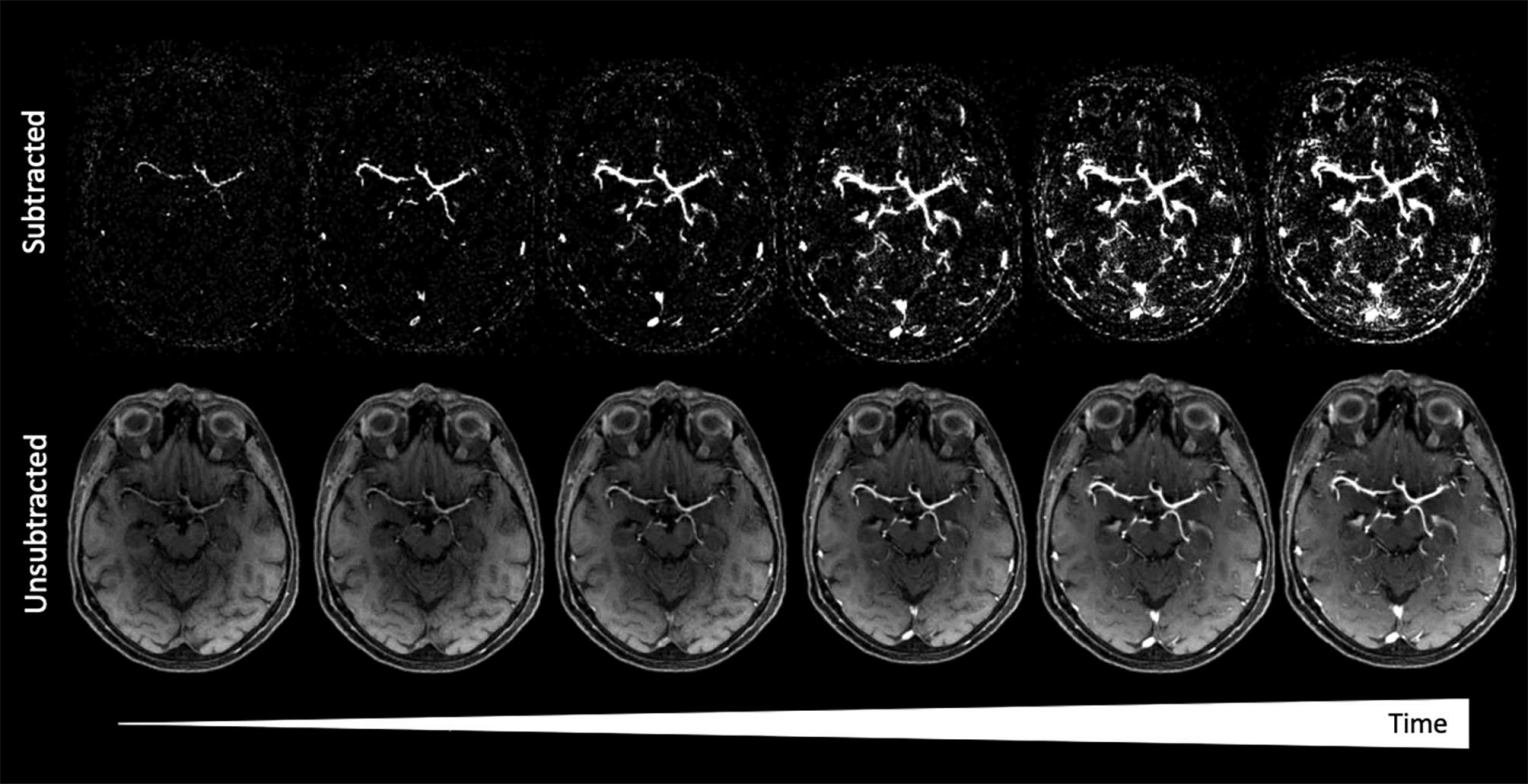
GRASP MRI from a 63-year-old woman imaged for headache reconstructed as time-resolved MRA images. Paired subtracted (top row) and source (bottom row) axial slices obtained from the 3D GRASP imaging volume at the level of the Sylvia cistern and fissure. Dynamic structural images reconstructed from 5 spokes of a golden-angle radial acquisition are shown, demonstrating the earliest arterial through the venous phases of gadolinium bolus passage.

**Figure 2:**
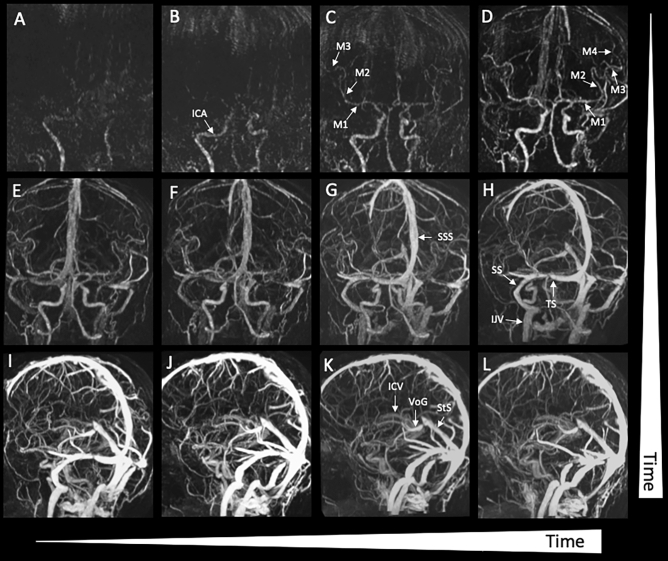
GRASP MRI from a 63-year-old woman imaged for headache reconstructed as time-resolved 3D MRA MIP images. Selected maximum intensity projections (MIPS) reconstructed from subtracted axial brain GRASP MRI using a 5 spoke reconstruction (**A**–**L**). 3D MIPs are rotated about the z-axis for illustrative purposes, exhibiting high-resolution, nearly 1–2 s resolved dynamic bolus passage through the cerebrovascular system, spanning earliest arterial through venous drainage phases. Arterial segments are labeled, including the internal carotid artery (ICA), middle cerebral artery (MCA) segments (M1, M2, M3, M4, and M5), and venous structures, including the superior sagittal sinus (SSS), transverse sinus (TS), straight sinus (SS), internal jugular vein (IJV), internal cerebral veins (ICV), vein of Galen (VoG), and straight sinus (StS).

Twenty examinations were evaluated by three subspecialty-certified neuroradiologists for image quality, vascular dynamic imaging/isolation of angiographic phases, and presence of reconstruction artifacts. Readers were instructed to identify peak arterial and venous enhancement phases, when sufficiently isolated, and to score the performance in 5 bilateral vascular segments including: (i) intracranial ICA; (ii) MCA-M1; (iii) MCA-M2; (iv) MCA-M3; v. MCA-M4; and vi. venous structures. A 4-point Likert scale was utilized, based on previously published criteria as follows: (0) severely limited, nondiagnostic examination; (1) fair, evaluation possible but limited; (2) good angiographic quality; and (3) excellent angiographic quality^25^. High temporal resolution native (i.e. standard structural) GRASP and subtracted MPR (Fig. 1) and subtracted, 3D rendered MIP angiographic reconstructions (Fig. 2), as well as the intensity-based gradient MPR images (see below) were made available to readers on the workstation running MeVisLab.

Raters reviewed images independently and subsequently generated a consensus result due to the complexity of the data structure. An initial test batch of three cases was compared to a contemporaneously acquired 3D TOF intracranial MRA to ensure that the rating methodology was harmonized across raters for each vessel segment of interest. While the concurrent availability of TOF-MRA and GRASP occurred in only a small subset of patients, the TOF in those subjects were used to establish a qualitative benchmark for standard-of-care arteriographic visualization and to ensure a uniform methodology among raters regarding vascular segments to be interrogated. Raters were further instructed to record the number of distinct arterial phases in all reconstruction algorithms, as well as the number of tissue/parenchymal capillary enhancement phases identifiable in the gradient image intended to accentuate hemodynamic signatures of the capillary phase.

In order to assess the potential influence of circulating blood volume (based on Nadler’s formula) and its consequent modulation of circulating gadolinium concentration using weight-based dosing, subject height and weight were recorded, and any interactions with angiographic quality were determined^26^.

### Statistical analysis

Categorical variables are reported as number and percent. Continuous variables were tested for normality using visual inspection of histograms and/or the Shapiro–Wilk test. When not normally distributed, continuous variables were reported as median and interquartile range (IQR). Intraclass correlation coefficients (ICC) were calculated using two-way mixed-effects models to determine consistency between 3 readers and assessed using published thresholds^27^. Spearman correlation was used to determine the association between vessel scores and other continuous variables. A two-tailed p-value of < 0.05 was designated for statistical significance. Statistical analysis was performed using Stata version 12.1 (StataCorp, College Station, TX).

## Results

Twenty patients without significant pathology identified on brain MRI were included in the final analysis from a cohort of 71 consecutive cases obtained at 3 T and with standard contrast dosing of 0.1 mmol/kg (subjects were excluded for tumor and/or postoperative changes n = 23, infection n = 15, inflammation/demyelination n = 10, and vascular malformation n = 3). Patient characteristics are summarized in Table 1. The median age of the cohort was 63 (IQR 32–73) with 15 (75%) female subjects. Using standardized contrast dosing, median contrast concentration was 6.9 (IQR 5.7–7.3). The most common indication for the brain MRI was evaluation for metastatic disease (70%), with other indications including headache, syncope, encephalopathy, and a suspected mass.

**Table 1:** Subject characteristics.

Subject characteristics (N = 20)	
Age, median (IQR)	63 (32–73)
Female sex, N (%)	15 (75%)
Height in meters, median (IQR)	1.6 (1.6–1.7)
Weight in kg, median (IQR)	69 (57–76)
Contrast concentration, median (IQR)	6.9 (5.7–7.6)
**Clinical indication, N (%)**	
Metastasis evaluation	14 (70%)
Headache	3 (15%)
Syncope	1 (5%)
Encephalopathy	1 (5%)
Head mass	1 (5%)

The ratings of performance for all vascular segments by the three neuroradiologists are summarized in Table 2. For native, structural GRASP imaging volumes without subtraction, the median rating across readers was 3 (IQR 3–3) indicating *excellent* angiographic quality, with ICC of 1.00 (95% CI 1.00–1.00), for all arterial and venous segments of interest. Median rating of subtracted images was 3 (*excellent*) for all arterial and venous segments, with the exception of a score of 2 (*good*) rendered for the MCA-M4 segments.

**Table 2:** Vascular segment rating and intraclass correlation between raters.

Reconstruction	Native grasp		Subtracted grasp		Hemodynamically-accentuated grasp	
Segment	Median consensus rating (IQR)	ICC (95% CI)	Median consensus rating (IQR)	ICC (95% CI)	Median consensus rating (IQR)	ICC (95% CI)
Intracranial ICA	3 (3–3)	1.00 (1.00–1.00)	3 (2–3)	0.95 (0.90–0.98)	2 (2–3)	1.00 (1.00–1.00)
MCA M1	3 (3–3)	1.00 (1.00–1.00)	3 (3–3)	1.00 (1.00–1.00)	3 (3–3)	1.00 (1.00–1.00)
MCA M2	3 (3–3)	1.00 (1.00–1.00)	3 (2–3)	1.00 (1.00–1.00)	3 (2–3)	1.00 (1.00–1.00)
MCA M3	3 (3–3)	1.00 (1.00–1.00)	3 (3–3)	0.80 (0.62–0.93)	3 (3–3)	0.93 (0.85–0.98)
MCA M4	3 (3–3)	1.00 (1.00–1.00)	2 (2–2)	0.94 (0.89–0.98)	3 (2–3)	0.91 (0.81–0.96)
Venous structures	3 (3–3)	1.00 (1.00–1.00)	3 (3–3)	1.00 (1.00–1.00)	3 (3–3)	1.00 (1.00–1.00)

*ICC* intraclass correlation coefficient, *CI* confidence interval, *IQR* interquartile range, *ICA* internal carotid artery, *MCA* middle cerebral artery.

**Figure 3:**
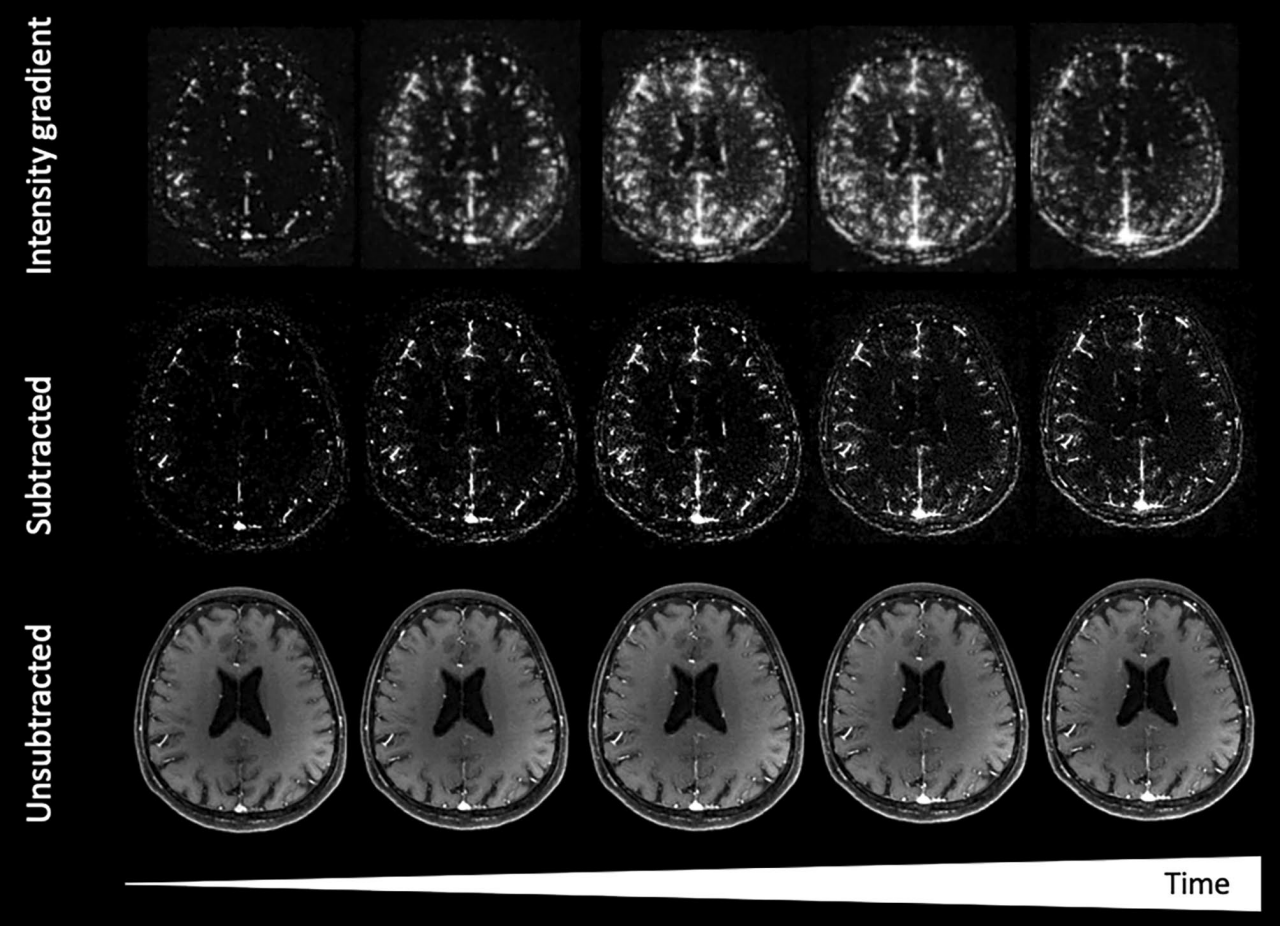
GRASP MRI from a 63-year-old woman imaged for headache reconstructed as time-resolved MRA images, including identifying parenchymal/capillary phase enhancement. Paired subtracted (middle row) and source (bottom row) time-resolved slices at the level of the corona radiata. Accompanying axial slices processed using voxel-wise intensity gradient (top row), as detailed in “Methods”, demonstrate accentuated parenchymal/capillary phase enhancement in both cerebral hemispheres, reflecting enhancing blood pool effects to improved advantage by comparison to standard subtracted or source structural images.

ICC ranged from 1.00 (95% CI 1.00–1.00) in the M1, M2, and venous segments (excellent reliability) to 0.80 (95% CI 0.62–0.91) for the M3 segment (good reliability). Median rating for temporal gradient images (Fig. 3) was 3 (excellent) including for the depiction of a capillary/parenchymal phase of enhancement and angiographic performance, apart from the cavernous/supraclinoid ICA, for which a median of 2 (*good*) was recorded. ICC for gradient images was 1.00 (95% CI of 1.00–1.00) for all vascular segments, apart from the M2 segment, which was 0.93 (95% CI 0.85–0.97), suggesting excellent reliability. Median height in meters was 1.6 (IQR 1.6–1.7) and weight in kg was 69 (IQR 57–73), with no significant correlation identified between vascular segment ratings and either subject weight, height, sex, or contrast dose (p > 0.05) to suggest a direct dependency of angiographic performance on circulating blood volume.

All reconstructions were found to have a median of 2 distinct arterial phases (IQR 1–3). A median of 2 distinct frames of capillary arterial phase (IQR 2–3) were found in the hemodynamic-accentuated temporal gradient images. No artifacts were reported as degrading evaluation, including either motion, ghosting, aliasing or other artifacts related to parallel acceleration, with the exception of a sub-optimal subtraction in a single case, which did not affect angiographic quality. Streak-like signal spikes related to radial k-space sampling were observed in the image periphery in the majority of cases without degradation of imaging or angiographic quality.

**Figure 4:**
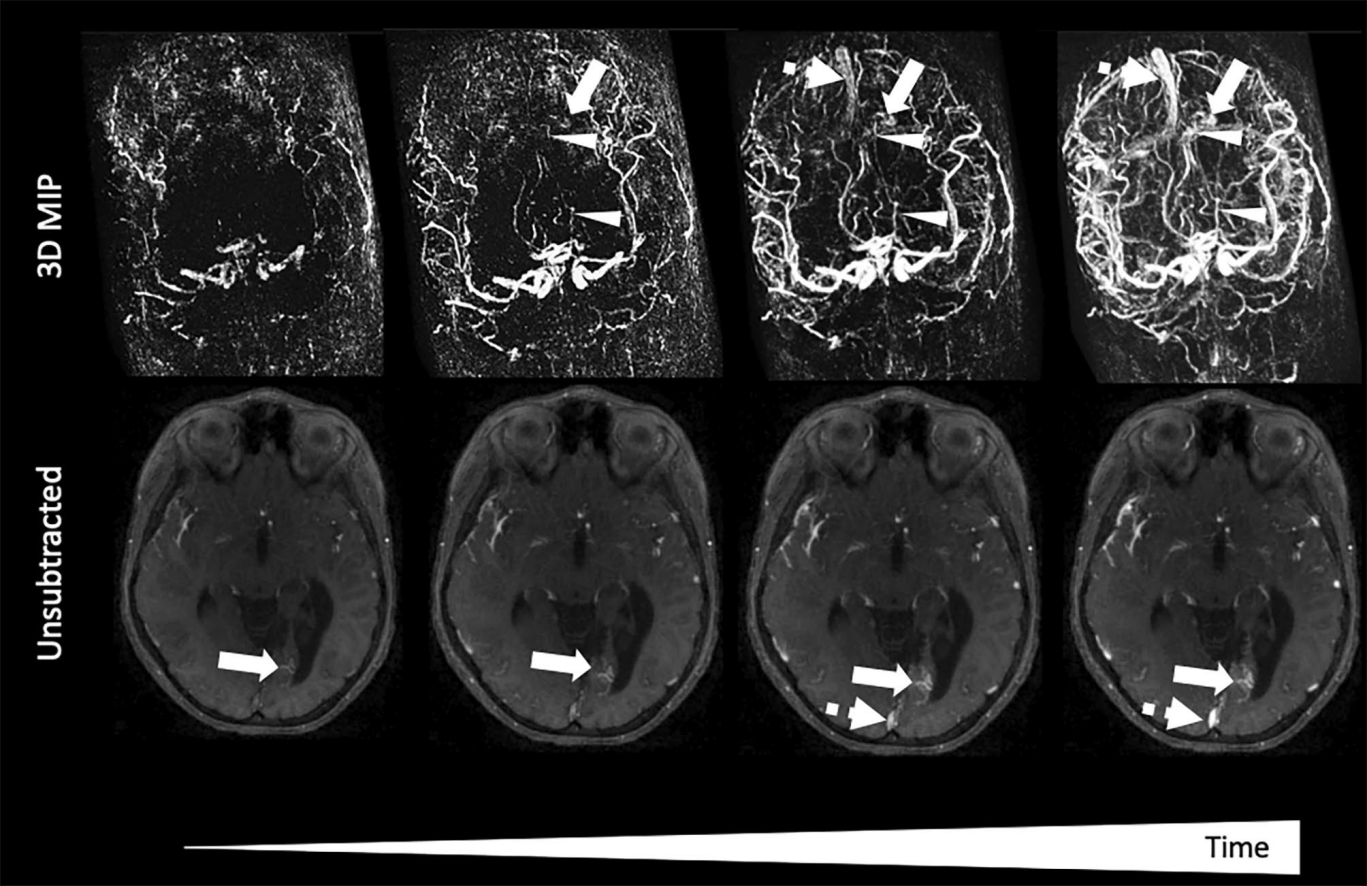
45-year-old man with history of parenchymal hemorrhage. Paired 3D maximum-intensity projection (MIP) and axial unsubtracted GRASP images demonstrate a small vascular nidus (arrow) supplied by the left posterior cerebral artery (arrowheads) and early filling of the superior sagittal sinus (dashed arrow) due to arteriovenous shunting.

**Figure 5:**
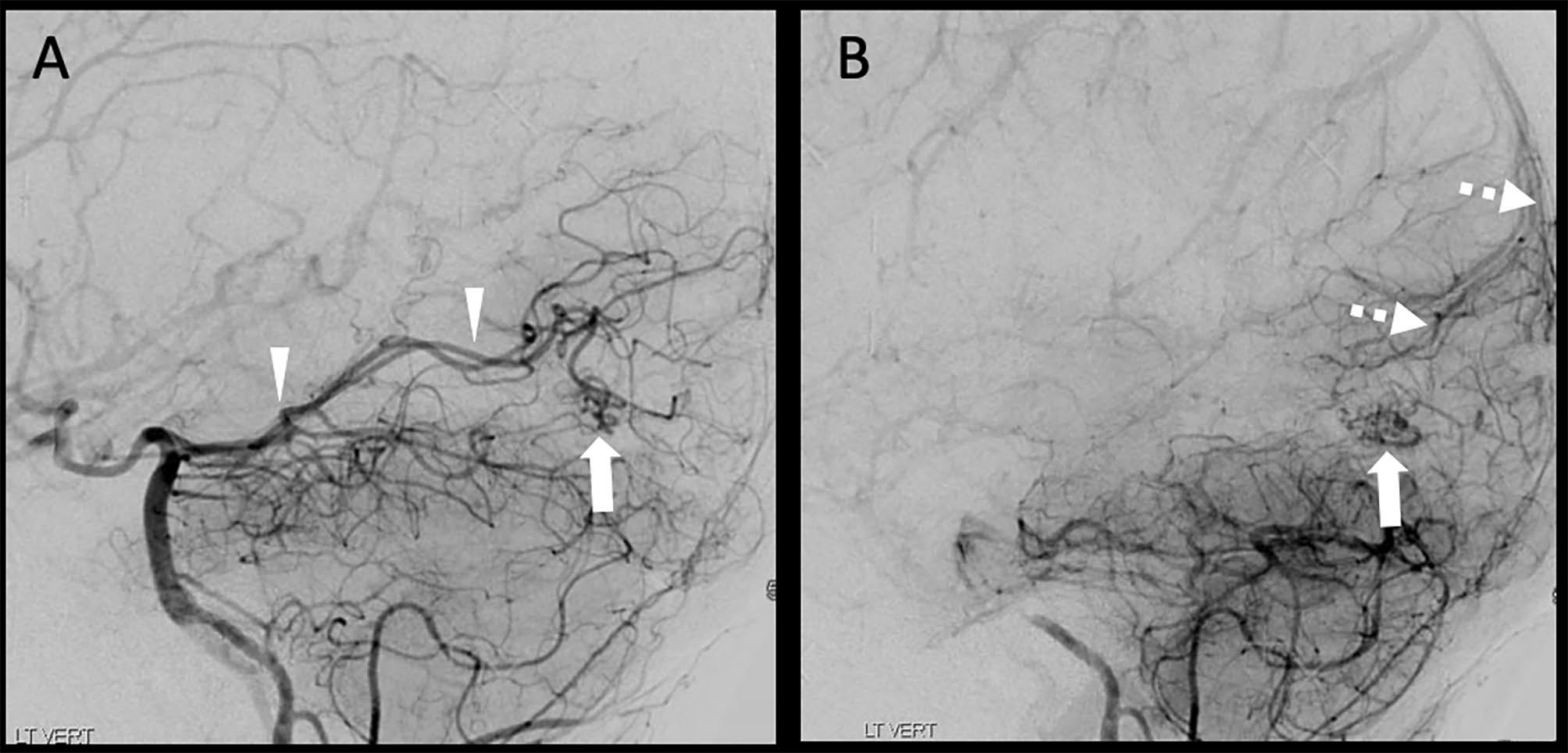
Lateral digital subtraction angiography from the same patient as Fig. 4 in the arterial (**A**) and early venous (**B**) phases demonstrating a small vascular nidus (arrow) supplied by the left posterior cerebral artery (arrowheads) and early filling of the superior sagittal sinus by cortical veins (dashed arrow) due to arteriovenous shunting.

A representative case of TR GRASP-MRA in the setting intracranial AVM (Figs. 4 and 5) demonstrates potential practical application of GRASP MRA to establish the presence or obliteration of residual arteriovenous shunting. GRASP MRA reconstructed post hoc and using routine structural scanning parameters clearly demonstrates the residual small nidus and arteriovenous shunting, which were confirmed on DSA.

## Discussion

Using an automated offline reconstruction and streamlined image processing pipeline, GRASP enables highly temporally-resolved dynamic angiography that can be obtained, even retrospectively, in routine practice without demand for further optimization of individual acquisition or timing parameters. Readily attainable 4D (i.e., time-resolved 3D) data sets were easily produced and yielded consistently good or excellent imaging in a three-rater review across both proximal and distally arborized cerebrovascular segments. The golden-angle acquisition scheme of GRASP proved particularly well-suited to this objective because images with the desired temporal footprint can be flexibly reconstructed post hoc and for any desired time point, allowing for excellent depiction of enhancement dynamics.

While other 4D dynamic MRA techniques have been previously developed, including highly accelerated non-cartesian techniques using golden angle sampling and compressed sensing, the flexibility of GRASP to be used as anatomical/structural sequence, producing pre- and post-contrast T1-weighted images, makes it highly suitable for routine application as reported here. The continuous radial stack-of-stars acquisition of k-space mitigates timing errors while motion and flow artifacts are inherently suppressed due to the robustness of the radial k-space trajectory^28^. After data acquisition, an arbitrary number of consecutive radial views can be combined into single temporal frames to create 4D angiography catered to specific applications, thereby relaxing the need for a priori determination of final temporal resolution. Even higher frame rates beyond the 1.58–2.07 s temporal resolution reconstructed in our study should be attainable simply by further reducing the number of radial spokes per volume, although our experience at those extremes remains limited.

Several existing techniques for TR-MRA, including TRICKS and TWIST, utilize center-weighted keyhole imaging while under-sampling the periphery of k-space^1–8^. However, view sharing may engender temporal smearing between ostensibly time-resolved volumes, confounding assessment for arteriovenous shunting. Signal-to-noise penalties inherent to conventional TR-MRA place additional constraints upon attainable spatial resolution, which in our experience limits its utility for small shunts. Compressed sensing methods like the approach used here can, by comparison, enable acceleration beyond the Nyquist limit by exploiting sparsity of the information content of the acquired data, although the suitability of such techniques for routine clinical use, despite promising performance, remains to be established^20–22^. Together with its performance as a structural brain imaging technique, GRASP is therefore uniquely adaptable for routine clinical workflow and general canvas for ad hoc dynamic imaging applications.

Several limitations to our study require discussion, including its retrospective nature. Importantly, however, the completely post hoc extraction of GRASP MRA in this cohort of brain imaging studies, all of which were obtained in routine practice for post-contrast structural T1-weighted imaging, underscores its versatility for dynamic imaging and secondary 4D angiography in a *real-world* setting. Similarly, sequence parameters were not modified or optimized for angiographic applications. In dedicate use as a prospective TR-MRA technique, optimizations including, for instance, greater flip angles to further accentuate vascular enhancement may be appropriate. Second, the standardized acquisition of our routine GRASP protocol in the axial plane introduces potential vascular inflow effects related to the steady state of the magnetization in the spoiled gradient-echo (fast low-angle shot, FLASH) scheme, in which non-saturated spins entering the inferior slices of the imaging volume may be hyperintense. This effect may be reduced through acquisition of sagittal 3D volumes as used commonly in other 3D brain imaging, or with use of non-selective excitations, although neither could be experimentally validated here due to the retrospective nature of the study. Nevertheless, subtraction of pre-contrast baseline images, which are similarly affected by inflow effects, provided excellent angiographic depiction and with faithful isolation of the arteriographic phase uncorrupted by inflow enhancement. The retrospective study design furthermore precluded any direct comparison between GRASP MRA and other angiographic techniques, with the exception of the limited initial comparison to TOF MRA during the reader calibration phase of the study. The number of vascular segments studied, including across multiple hemodynamic phases and using multiple reconstruction/rendering modes all in an entirely custom post-processing and visualization pipeline, also precluded a truly independent methodology for the reader ratings. However, as reported in similarly designed past studies, a consensus read from three subspecialized neuroradiologists represents a reasonable and generalizable initial experience from which to study the technique^2,29,30^. To limit variability, the current study analyzed exams only at 3 T and only using a single high-relaxivity contrast agent (gadobutrol). Imaging at lower field strength or with contrast agents exhibiting weaker relaxation enhancement could impact angiographic quality in non-trivial ways. In this respect, further exploration across varying imaging conditions is necessary to ensure generalizability^25,31^. The long calculation times currently required for post hoc GRASP reconstructions at extremely high temporal resolution, which can take several hours, presently pose a challenge for widespread use in acute settings. However, we anticipate that significant acceleration of the reconstruction can be achieved by using dedicated graphics processing units (GPUs), by utilizing cloud computing resources, or by using Deep Learning-based techniques, which are being explored by our group for future integration^32^. Similarly, the image postprocessing and visualization steps presently require direct user involvement in our pipeline, but can be shortened or potentially automated with tailored and optimized software tools. Most importantly, the actual diagnostic utility and accuracy of 4D GRASP MRA was not studied, and dedicated investigation in disease-specific applications is therefore necessary to establish its full diagnostic performance.

In conclusion, GRASP MRI obtained as part of routine clinical care can be simultaneously or even retrospectively utilized for production of 4D MRA with high spatial and temporal resolution, reliably demonstrating excellent angiographic quality and a low tendency for angiographic artifacts. The streamlined nature of the acquisition and the flexibility for post hoc angiographic reconstruction make it an attractive option for potential clinical TR-MRA. Further exploration into diagnostic accuracy in disease-specific applications is warranted.

## Data Availability

The datasets generated and/or analyzed during the current study are not publicly available due to reasons of sensitivity (human data) but are available from the corresponding author on reasonable request.
